# Beyond the Bench: Problems along the New “Silk Road”

**DOI:** 10.1289/ehp.112-a879

**Published:** 2004-11

**Authors:** Andrea M. Hricko

The inexpensive sweater you buy in New Jersey, the bargain dresser you buy in Wisconsin, and the low-cost toys you buy in Ohio all have one thing in common: most likely they were made in Asia, and chances are they entered the United States through a Southern California port. These consumer goods may be easy on the pocketbook, and thriving international trade may be good for the economy, but the environment is seeing some serious impacts. Today the NIEHS-funded Southern California Environmental Health Sciences Center (SCEHSC) is working to raise awareness about these impacts. The center is a partnership between investigators at the University of Southern California and the University of California, Los Angeles.

The adjacent twin ports of Los Angeles and Long Beach have become the world’s third largest port complex, flooding the region with imported cargo containers, which are transported by big-rig diesel trucks and by ships and freight trains operating on low-grade fuel. The booming trade has made the ports the region’s number-one source of air pollution. And the ports keep growing, creating a need for expanded freeways and larger intermodal facilities where containers are moved from trucks onto trains.

Emissions from foreign-flagged ships (nearly all ships entering the two ports) are currently unregulated, constituting a major concern for residents in the port communities. Residents along truck-congested freeways and rail routes are also impacted. “We have homes and parks right next to the rail yard,” says Angelo Logan, director of the grassroots organization East Yard Communities for Environmental Justice, “and sometimes locomotives idle here for hours.”

The SCEHSC, spurred by community concerns raised at a 2001 Town Meeting it sponsored, has been dedicating significant attention to these global trade impacts. Through the center’s Community Outreach and Education Program, center investigators and outreach staff are raising awareness of the need to reduce trade-related air pollution and better protect residents from the surge in such pollution.

Center investigators outreach staff have present ed data to elected officials on air pollution’s respiratory effects on children. Among these are the results of the 10-year longitudinal Children’s Health Study (directed by SCEHSC director John Peters), which shows a larger propor tion of children in more polluted communities suffering lung function deficits, with lifelong health significance. Staff have also testified on the health effects of exposure to diesel exhaust at hearings to ban idling of diesel trucks at the ports, to triple the capacity of a freeway that goes through dozens of low-income, primarily Latino communities, and to construct huge trucking distribution centers in Riverside, one of the country’s most polluted cities.

Center member Ed Avol serves as an appointee to the Mayor of Los Angeles’ special task force on port emissions, which was convened in October 2004. The center recently published a policy brief looking at the need to place a higher priority on health when key development decisions are made—for example, in regards to expansion of ports, rail freeways, or warehouses. And center member John Froines and outreach director Andrea Hricko discussed the impacts of global trade on local communities at the November 2003 roundtable discussion “Globalization, International Trade, and Environmental Health,” sponsored by the Institute Medicine of the National Academies.

Center staff are also working directly with community members, for example by taking high school and college students on tours of the ports and teaching them how to take ultrafine particle measurements. The center is also cosponsoring a Town Meeting in February 2005 with a theme of “Local and Regional Health Impacts of the Ports and Global Trade.”

## Figures and Tables

**Figure:**
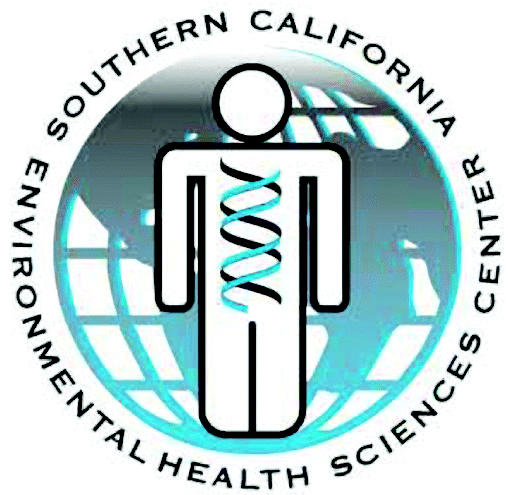


**Figure:**
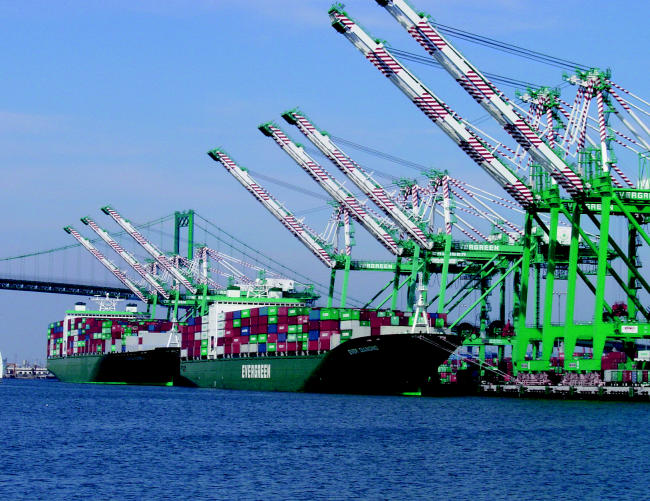
**The high price of cheap goods.** Container ships bring inexpensive goods from around the world into the Port of Los Angeles, but the pollution caused by transportation of these goods by ship, truck, and train has human health costs.

